# Role of Resistance to Innovation, Lack of Intercultural Communication, and Student Interest on the Student Demotivation Results Towards the English Education System

**DOI:** 10.3389/fpsyg.2022.922402

**Published:** 2022-07-01

**Authors:** Jin Wang, Lei Pan

**Affiliations:** ^1^Department of Foreign Languages, Xinzhou Normal University, Xinzhou, China; ^2^School of International Education, Hainan Medical University, Haikou, China

**Keywords:** resistance to innovation, lack of intercultural communication, students' interest, students' demotivation, English education system

## Abstract

Recently, second-language learning success depends upon the students' interest and motivation by adopting innovations that require regulators' and new researchers' emphasis. Hence, this article explores the role of resistance to innovation, lack of intercultural communication, and students' interest on the students' demotivation. The article also examines the mediating role of students' demotivation among resistance to innovation, lack of intercultural communication, lack of students' interest, and failure of the English education system in China. This study has gathered the data using survey questionnaires and analyzed the collected data using smart-PLS. The results exposed that the resistance to innovation, lack of intercultural communication, and students' interest have a significant and positive linkage with students' demotivation. The findings also indicated that students' demotivation significantly mediates among resistance to innovation, lack of intercultural communication, lack of students' interest, and failure of the English education system in China. This study guides the policymakers to develop the policies related to improving the English education system in China using innovation that enhances students' interest and motivation.

**JEL Classifications:**

O31, O32, H75.

## Introduction

The rapid technological changes have closed the distances between different cultures of the world. As a result of this globalization, cultural exchange has taken the place. One of the common barriers reported as a result of this cultural exchange is communication. Every country has its own language. There is no common language in the world. The developing countries are in need to learn the developed countries' languages to commutate. With the passage of time, the language rated as common in the world is English. The phenomenon of English medium instruction (EMI) in higher education is now well-established and spreading rapidly over the world. Many academic fields are becoming more universal in their use of English, and much higher education (HE) institutions are implementing “Englishization” of their curricula to achieve internationalization. English has evolved from being taught as a foreign language alongside other disciplinary-focused courses to being an essential educational language used for studying and teaching non-language-related academic subjects as a result of this change in the medium of instruction. Literature proposed that English is narrated as an international language (McKay, 2018; Xu, 2018). The developing countries not having English as their native language pay special attention to English learning in order to get closer to the world. China is one of the fastest growing countries on the globe. Not only the world but China is also attracted to the world in terms of business, education, etc.

**Figure 1:**
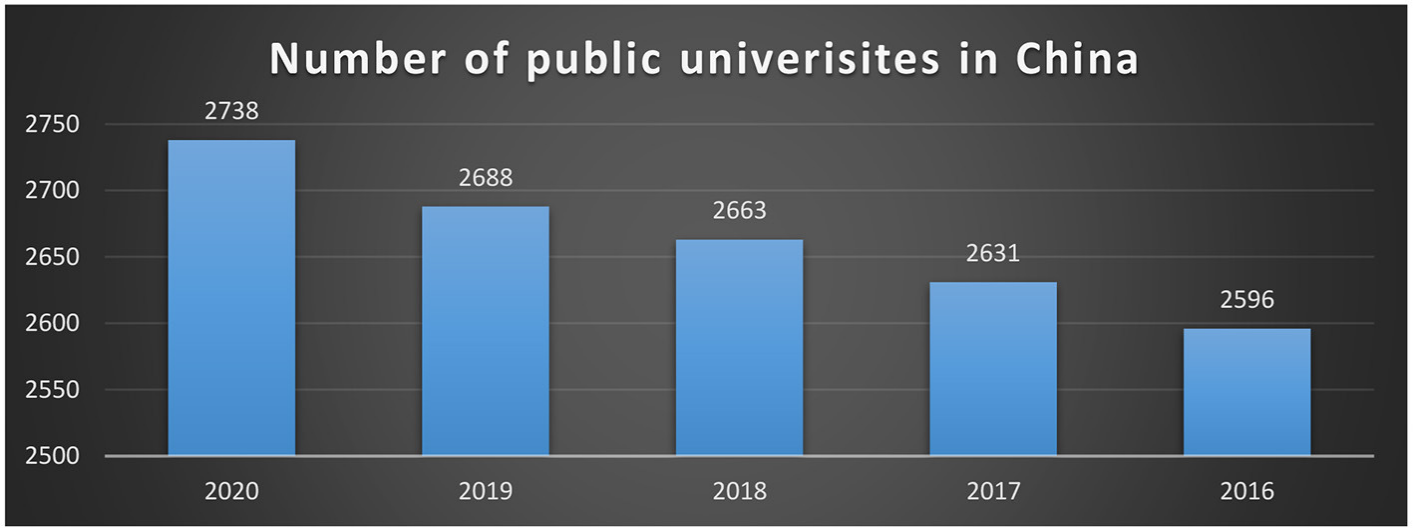
Number of public universities in China.

English language education has been a priority in China for the last quarter-century, and competence in English is widely recognized as a national as well as individual benefit. On a national level, the Chinese government sees English language education as playing an important role in national modernization and growth. Although the rationales articulated in a series of policy statements for expanding and strengthening English language teaching (ELT) in the education system have changed over time in response to perceived national development priorities, the importance and benefits of national proficiency in the language have never been questioned (Ma et al., 2021; Wang and Fan, 2021). On the individual level, English proficiency may lead to a plethora of economic, social, and educational prospects, that is, it can offer access to both material and “symbolic capital” for the improvement of one's wellbeing. It can be a passport to further education in the United States or overseas, lucrative work in the public or private sector, professional progress, and social status, for example. Because of the importance of English and the growing demand for English competence, enormous public and private efforts and resources have gone into English language instruction. The number of public universities in China is given in Figure 1.

This study addresses some gaps that exist in the literature like (1) being one of the important topics like English education system and capital and individual performance although researched although but still not reached its peak, (2) Fomenko et al. (2020) investigated the barrier of intercultural communication, whereas the present student has employed number of variables like resistance to innovation, students lack of interest, students demotivation and English language and check the model in Chinese educational institutions, (3) Badrkoohi (2018) investigated the association between demotivation and intercultural communication, whereas this study adds the variable and also checks the mediation effect in Chinese perspective, (4) Saeed et al. (2018) worked on the students interest factors, whereas this study adds the variables like resistance to innovation, students demotivation, students demotivation, and English language and checks the model with new data set; this study checks the model in Chinese perspective with new data set, (5) Kusdemir and Bulut (2018) worked on the student motivations, whereas this study takes the students' demotivation and tests the model in Chinese educational instructions, and (6) a study by Schröer (2021) also examined the innovation role on the education system and suggested that the upcoming articles should add other factors to predict education system, and this article uses the resistant to innovation and lack of intercultural communication to predict the English education system. The significances of this study are as follows: (1) it highlights the importance of intercultural communication and education system in countries where the native language is not English like Chinese, (2) helps professional to revamp their policies for the betterment of linguistic education like English in China, and (3) helps the researchers to identify the linguistic importance for any country not having English as their mother language.

The study structure is divided into five phases. The first phase presents the introduction. In the second phase of the study, the pieces of evidence regarding resistance to innovation, lack of intercultural communication, lack of student interest, student demotivation, and failure of the English education system are discussed in the light of past literature. The third phase of the study shines the spotlight on the methodology employed for the collection of data regarding resistance to innovation, lack of intercultural communication, lack of student interest, student demotivation, and failure of the English education system and its validity is analyzed. In the fourth phase, the results of the study are compared with the pieces of evidence reviewed from the literature. In the last phase, the study implications along with the conclusion and future recommendations are presented, which concludes the article.

## Literature Review

The change is not accepted with a whole heart almost in aspects of society. Either it is business or educational institution, the employees or students usually express resistance to the adoption of the change. Resistance is one of the major causes of failure in innovation, and this resistance demotivated the students who want to make some innovations. In this context, Talwar et al. (2020) and Tang and Chen (2022) checked the relationship between resistance to innovation and student demotivation. In this article, we focused on digital innovation and mentioned that resistance occurs due to poor academic learning of students. Search protocols and research time period was 2002–2020. In this article, it is further added that the rate of failure is too high in 2009 in China. In conclusion, the article addresses the issue of failure such as consumer resistance, and this resistance loses the hope of students. Innovation launching is a process in which an uncertain outcome has been received. This outcome may be positive or negative, people avoid it due to the risk of a negative outcome, and this negative mindset demotivated the students who want to make or want to be part of innovation. In this context, Joachim et al. (2018) checked the relationship between restraining innovation and its effect on student hope. For this article, the method of AIR typology is used in Germany for conducting the result. This article is based on a study in the period of 1981–2018. In conclusion, the article addresses the problem related to innovation and called that resistance to innovation is a big reason for student demotivation. Investment in the education sector is considered a key element in human development. The World Bank has committed has innovation in the educational sector will reduce the poverty and provide political stability, But unfortunately, these innovations are resisted in Pakistan. Khushik and Diemer (2018) checked the relationship between innovation and students' demotivation. When the innovation does not bring in education, then students get demotivated to learn and explore the research. The main resistance is the spending of less amount of GDP in the education sector. Many rules have been made from 1971 to 2018, but nothing improved. In conclusion, the article addressed that, due to no innovation in the education sector, students become hopeless and have no chance other than to learn according to the older education system. Thus, the literature proposed that change in terms of innovation usually faced resistance (Choi et al., 2019; Senbeto et al., 2021). Thus, the following hypothesis is derived from the above debate:

**H1:** Resistance to innovation has a positive and significant impact on student demotivation in China.

The recent globalization brings the world closer. The people of developing countries are interested to approach developed countries with intentions for higher studies, business, etc. Cultural communication plays a vital role while cultural exchange in terms of education, business, etc. In this cultural exchange, one of the major barriers between developed and developing countries is communication. Literature witnessed that communication is the barrier (Apriyanti, 2018; Grujić and Krneta, 2018; Chien et al., 2021c). The students of developing countries who chose developed countries for education faced communication barriers. The developed nations communicate in their native language. Thus, the student approaches linguistic institutions to overcome it, but due to scarcity of resources like the less skilled coaching staff and lack of modern technology, the students get demotivated. In this context, Donnery (2022) checked the student demotivation factors in the English language and proposed that factors like overcrowded classes and the learning environment cause student demotivation. Thus, the international community requires their language for all sorts of communication, and students get demotivated as a result of different factors. In addition, a study by Badrkoohi (2018) also investigated the association among intercultural communication and student demotivation and revealed that the intercultural communication has a positive and significant impact on student demotivation. Thus, the following hypothesis is derived from the above debate:

**H2:** Lack of intercultural communication has a positive and significant impact on student demotivation in China.

There are number of factors faced by the students during their study, e.g., the student choice factor strongly affects the student results. Most of the times, the students lack interest in education due to multiple factors like the unskilled coaching staff, institution environment, force studies, etc. (Saeed et al., 2018; Indawati, 2021). Once the student lacks interest, it affects one's motivation. The literature proposed that there is an association between the student's interest and motivation or demotivation (Kusdemir and Bulut, 2018; Dingess II D. R., 2020; Chien et al., 2021a). In this context, Dingess II D. R. (2020) explored the nexus between students' interest and motivation through a literature review. The study concluded that the student's interest strongly influences the student's motivation or demotivation. A student who studies with full interest results in getting motivation and produces good results otherwise *vice versa*. Similarly, Renninger et al. (2018) also explored whether there is any relationship between motivation and interest and are of the view that motivation and interest are significantly associated. Thus, the following hypothesis is derived from the above debate:

**H3:** Lack of student interest has a positive and significant impact on student demotivation in China.

One of the common differences between developed and developing countries is the education system, as well as quality. English is the world language. The developing countries having English not their native language faced a number of international communication barriers, which sometimes ruin their efforts. The education sector of any country is the backbone of the country's development, and the students are the blood of these institutions. Globalization urges the students to pursue higher studies in developed economies in order to learn advanced education tools to compete in the world. One of the common barriers faced by countries having not English as their native language is communication (Fomenko et al., 2020; Gardner, 2020). Thus, the students approach the local linguistic schools. Due to numerous factors, the students get demotivated, which results in poor results. The literature also proposed that demotivation leads to poor performance of students (Ghafournia and Farhadian, 2018; Pincay et al., 2019; Szabo et al., 2020). This poor performance of the students affects the collapse of the entire education system. Thus, there is an association between students' demotivation and the education system of any country. Thus, the following hypothesis is derived from the above debate:

**H4:** Student demotivation has a positive and significant impact on failure in English education system in China.

A motivated student leads to career success, but in the case of demotivation, it is *vice versa*. The students get motivated by a number of factors. Literature proposed that the student's motivation affect the students' performance. The students get motivated or demotivated due to a number of factors like educational institution factors, innovation, change factors, etc. One of the common and major factors, which affect the student's motivation, is change. The acceptance of change is one of the difficult factors as per psychological literature (Province, 2018; Chien et al., 2021b; Xu and Ma, 2021). In terms of education, this change can be in different forms like the introduction of technology in the education sector, the new curriculum, etc. The students resist the change, and as a result, they get demotivated. The association between motivation and resistance to innovation is proposed in the literature (Fischer et al., 2019; Chen et al., 2020). The student's demotivation affects the entire education system of the nations in terms of bad results. Thus, demotivation influences the nexus between innovation resistance and education system failure. Therefore, the student's demotivation can be employed as a mediator as supported by the literature (Zhang et al., 2020; Sun et al., 2021). Thus, the following hypothesis is derived from the above debate:

**H5:** Student demotivation significantly mediates between resistance to innovation and failure of the English education system.

The education system of any country is the facilitator of the country's development in multiple terms. A better education system will produce good and skilled individuals who will support their nations to match the international standards. Thus, the failure or success of the country's education system decides the country's progress in the world. The world has become a global village, and cultural exchange is at its peak. In this global village, intercultural communication is acting as a bridge (Shvachkina and Rodionova, 2018; Lobanova et al., 2019). The countries not having English as their native language face hurdles in intercultural communication. In this study, the education system of the country plays a vital role. The countries not having good linguistic institutions results in a week of intercultural communication. The students approach these linguistic institutions and result in getting demotivated. Thus, the student's demotivation gets affected by intercultural communication and affects the country's education system due to weak performance (Badrkoohi, 2018; Kurosh and Kuhi, 2018). In the presence of literature, the student's demotivation can be employed as a mediator as supported by the literature (Zhang et al., 2020; Sun et al., 2021). Thus, the following hypothesis is derived from the above debate:

**H6:** Student demotivation significantly mediates between the lack of intercultural communication and failure of the English education system.

We are living in a digital world. In this study, the countries that failed to meet the world standards are crushed by the rising competition, the only factor that enables the country to respond to the world. The success or failure of the country's education system is based on the student's performance. The student's performance is further based on their motivation as literature proposed that there is an association between students' performance and motivations (Mahler et al., 2018; Bosch et al., 2021). In contrast, the student's performance is based on the student's interest (Crouch et al., 2018; Lawner et al., 2019). Thus, the student's interest affects the education system of the country. Therefore, the student's demotivation affects the education system and is affected by the students' interests. Furthermore, in the presence of literature, the student's demotivation can be employed as a mediator as supported by the literature (Zhang et al., 2020; Sun et al., 2021). Thus, the following hypothesis is derived from the above debate:

**H7:** Student demotivation significantly mediates between lack of student interest and failure of the English education system.

## Methodology

The article explores the role of resistance to innovation, lack of intercultural communication, and students' interest in the students' demotivation and also examines the mediating role of students' demotivation among resistance to innovation, lack of intercultural communication, lack of students' interest, and failure of English education system in China. This study has gathered the data using survey questionnaires. The questionnaire is extracted from past studies; the lack of intercultural communication (LIC) has seven items extracted from Ay et al. (2018), resistance to innovation has eight items taken from Hosseini et al. (2016), lack of student' interest (LSI) has four items extracted from Zhoc et al. (2019), students' demotivation (SDM) has seven items taken from Afthanorhan et al. (2019), and failure of English education system (FEES) has five items taken from Jääskeläinen et al. (2022). These items with sources are given in Table 1.

**Table 1:** Questionnaire of the study.

**Items**	**Statements**	**Sources**
**Lack of intercultural communication**		
LIC1	“I am tense and nervous while interacting with people from different cultures.”	Ay et al., 2018
LIC2	“Engaging in a group discussion with people from different cultures makes me nervous.”	
LIC3	“While participating in a conversation with a person from a different culture, I get nervous.”	
LIC4	“Ordinarily, I am trepidations and nervous in a conversation with a person from a different culture.”	
LIC5	“I am afraid to speak up in conversations with a person from a different culture.”	
LIC6	“My thoughts become confused and jumbled when interacting with people from different cultures.”	
LIC7	“Communicating with people from different cultures makes me feel uncomfortable.”	
**Resistance to innovation**		
RTI1	“I will wait to adopt new technology until it proves beneficial.”	Hosseini et al., 2016
RTI2	“I need to clarify some queries and justify the reasons to adopt new technology.”	
RTI3	“I am waiting for the right time and required capability to adopt new technology.”	
RTI4	“I fear wasting my time using new technology.”	
RTI5	“I need to get a solution for some of my complaints and objections before I adopt new technology.”	
RTI6	“I fear certain changes in the organization may impose on me.”	
RTI7	“Innovation is not for me.”	
RTI8	“It is unlikely that I will adopt innovation in the near future.”	
**Lack of student interest**		
LSI1	“I do not regularly work with other students on course areas where I have problems.”	Zhoc et al., 2019
LSI2	“I am not regularly get together with other students to discuss courses.”	
LSI3	“I am not regularly studying with other students.”	
LSI4	“I do not feel part of a group of students committed to learning.”	
**Student demotivation**		
SDM1	“I am not satisfied with the quality of service provided by my institution.”	Afthanorhan et al., 2019
SDM2	“I am not satisfied with the collection provided by the institution.”	
SDM3	“I am not satisfied with the facilities provided by the institution.”	
SDM4	“I am not satisfied with the environment provided by the institution.”	
SDM5	“I will not recommend my friends to make full use of this institution.”	
SDM6	“I will continue using this institution.”	
SDM7	“In general, I am not satisfied with this institution.”	
**Failure of the english education system**		
FEES1	“I am not engaged with the education provided by the institution.”	Jääskeläinen et al., 2022
FEES2	“I am not satisfied with the education provided by my institution.”	
FEES3	“I am not interested in the education provided by the institution.”	
FEES4	“I am not motivated to gain this education in the same institution.”	
FEES5	“The education is unattractive for me that I gain from this institution.”	

**Figure 2:**
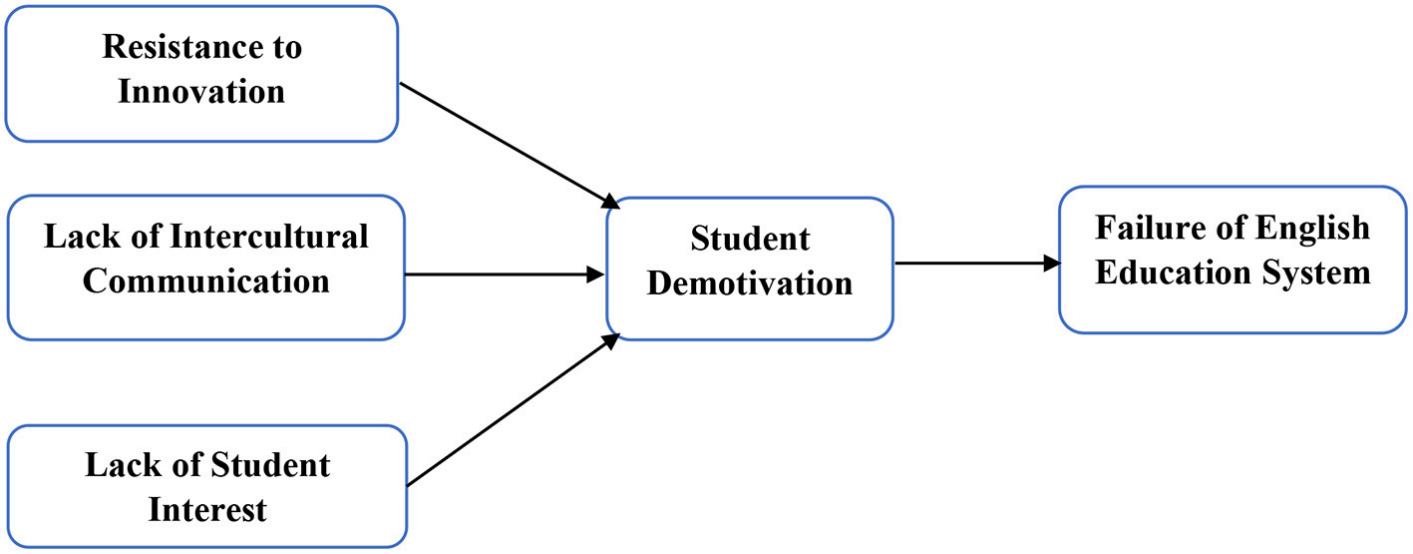
Theoretical framework.

In this article, the resistance to innovation, lack of intercultural communication, and lack of students' interest have been used as independent variables, while students' demotivation has been taken as mediating variables, and the failure of the English education system has been used as a predictive variable. The questionnaires have been sent to the respondents using personal visits. The students who are learning English as the second language at educational institutions are the respondents of the study. They are selected based on purposive sampling. The researchers have sent around 623 surveys to the English learning students in the Beijing, China. This study has selected the education sector in Beijing, China, because most of the educational institutions exist there. In addition, researchers have received only 375 surveys after 1$\frac{1}{2}$ months. These surveys have around 60.19% response rate. In addition, the smart-PLS was used to analyze the primary data. It is an effective statistical tool that effectively estimates the primary data, even in complex frameworks and large sample sizes (Hair Jr et al., 2021). This study has employed the innovation adoption theory that exposed that innovation brings the changes in the process and enhances the efficiency of the business and *vice versa*. This article also examines the resistance to innovation role on the failure of education system and exposes that if the institution resists to adopt innovation, then it fails to achieve their goals. Based on this theory, the framework has been developed and is illustrated in Figure 2.

## Findings of the Study

This article's results have tested the reliability using composite reliability and alpha, and the statistics exposed that reliability is significant because the values are larger than 0.70. In addition, the results have also tested the content validity using factor loadings, and the statistics exposed that content validity is valid because the values are larger than 0.50. Finally, this article's results have also tested the convergent validity using average variance extracted (AVE), and the statistics exposed that convergent validity is valid because the values are larger than 0.50. Table 2 shows the convergent validity of the study.

**Table 2:** Convergent validity.

**Constructs**	**Items**	**Loadings**	**Alpha**	**CR**	**AVE**
Failure of English education system	FEES1	0.707	0.884	0.916	0.686
	FEES2	0.808			
	FEES3	0.897			
	FEES4	0.861			
	FEES5	0.857			
Lack of intercultural communication	LIC1	0.821	0.888	0.912	0.602
	LIC2	0.897			
	LIC3	0.826			
	LIC4	0.861			
	LIC5	0.610			
	LIC6	0.703			
	LIC7	0.667			
Lack of student interest	LSI1	0.851	0.803	0.871	0.629
	LSI2	0.784			
	LSI3	0.806			
	LSI4	0.725			
Resistance to innovation	RTI1	0.891	0.947	0.956	0.732
	RTI2	0.880			
	RTI3	0.854			
	RTI4	0.928			
	RTI5	0.877			
	RTI6	0.812			
	RTI7	0.832			
	RTI8	0.758			
Student demotivation	SDM2	0.834			
	SDM3	0.822	0.868	0.901	0.604
	SDM4	0.767			
	SDM5	0.782			
	SDM6	0.757			
	SDM7	0.692			

This article's results have tested the discriminant validity using the Heterotrait Monotrait (HTMT) ratio. The statistics exposed that the discriminant validity is valid because the values are not larger than 0.90. Table 3 shows the results of the HTMT ratio.

**Table 3:** Discriminant validity.

	**FEES**	**LIC**	**LSI**	**RTI**	**SDM**
FEES					
LIC	0.621				
LSI	0.728	0.539			
RTI	0.828	0.503	0.521		
SDM	0.540	0.449	0.592	0.427	

**Figure 3:**
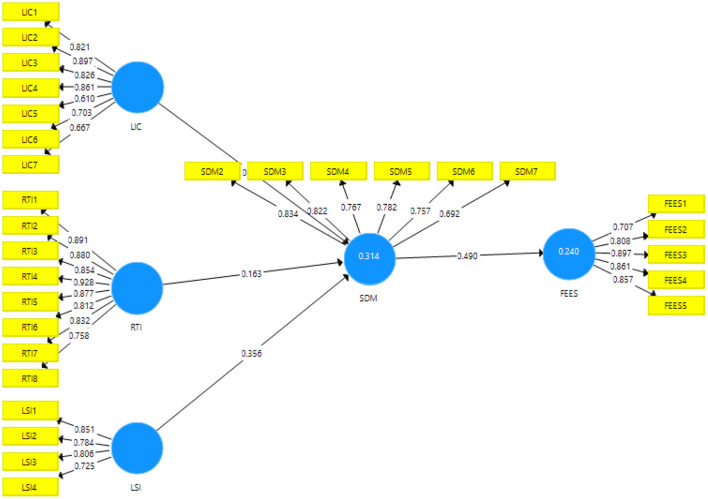
Measurement model assessment.

**Figure 4:**
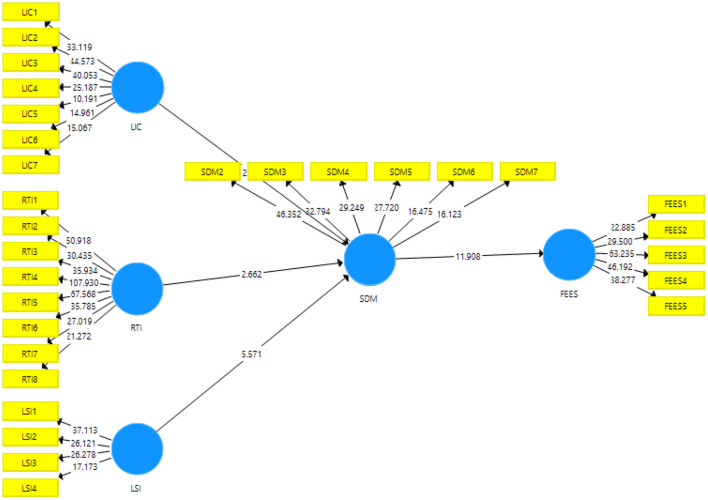
Structural model assessment.

The results of the direct path exposed that the resistance to innovation, lack of intercultural communication, and students' interest have a significant and positive linkage with students' demotivation and accept H1, H2, and H3. Table 4 shows the direct association among variables. Figure 3 shows Measurement model assessment and Figure 4 shows Structural model assessment.

**Table 4:** Direct path.

**Relationships**	**Beta**	**Standard deviation**	**T statistics**	***P***	**Lower limits**	**Upper limits**
LIC -> SDM	0.165	0.058	2.868	0.005	0.059	0.265
LSI -> SDM	0.356	0.064	5.571	0.000	0.254	0.487
RTI -> SDM	0.163	0.061	2.662	0.009	0.023	0.251
SDM -> FEES	0.490	0.041	11.908	0.000	0.405	0.558

The findings of the indirect path indicated that students' demotivation significantly mediates among resistance to innovation, lack of intercultural communication, lack of students' interest, and failure of the English education system in China and accepts H4, H5, and H6. Table 5 shows the indirect linkage among the variables.

**Table 5:** Indirect path.

**Relationships**	**Beta**	**Standard deviation**	**T statistics**	***P***	**Lower limits**	**Upper limits**
LIC -> SDM -> FEES	0.081	0.028	2.890	0.005	0.030	0.133
LSI -> SDM -> FEES	0.174	0.038	4.528	0.000	0.104	0.255
RTI -> SDM -> FEES	0.080	0.033	2.435	0.017	0.012	0.135

## Discussions

The results indicated that resistance to innovation has a positive relation to students' demotivation. These results agree with Moreno-Guerrero et al. (2020), which posits that just like in other fields of life, in education, innovation has great significance. The adoption of innovative sources of learning allows the students to keep in contact with their tutors all the time and make connections with their fellow students or seniors. These connections help them attain knowledge at any time and thus, create learning motivation in students. But, the students are not provided with innovative means of learning. These results are also supported by Chien (2019), which proclaims that the innovative means of learning used in the classrooms or outside help develop essential learning skills like organization, responsibility, independent work, initiative, collaboration, self-regulation, and self-confidence. But, in case the students are unable to benefit from learning innovation, there is a lack of learning skills, and they are demotivated to learn the subject. Hence, resistance to innovation causes student demotivation.

The results showed that lack of intercultural communication has a positive relation to students' demotivation. These results agree with Yunus (2018), which examines the role of lack of intercultural communication in students' demotivation. The study shows that when in a society, people of different cultures live together, and events are conducted where they may have the chance to meet, share their ideas, and develop common values; intercultural communication enhances the students' information and develops an interest in them to learn more about one's own and others' cultures. Moreover, the lack of intercultural communication reduces the student's motivation to learn more. These results are also in line with Chen (2018), which claims that when the students do not experience intercultural communication, they are unable to learn about one another's living styles. This does not allow developing the urge and stamina of the students to try to understand and learn more aspects of the living style of the party in front. So, the lack of intercultural communication leads to students' demotivation. The results revealed that students' lack of interest has a positive relation to students' demotivation and matched with those suggested by Cao and Meng (2020), which shows that it is the interest in the students which motivates them to follow a timeline, accomplishes the tasks assigned to them, focuses on the subject, develops understanding, solves learning problems, and attains comprehensive knowledge. But, when the students do not have enough interest, they are unable to act upon the syllabus and perform efficiently during classes. Thus, English education that is totally based on student interest and motivation is feared to meet with failures.

The results also stated that student demotivation is a mediator between resistance to innovation and failure of the English education system. These results are supported by Guan et al. (2020), which shows that when there is resistance to the adoption of innovation in education, as the lack of the use of different technologies, digital devices, software, and online learning portfolios, it restricts the scope of the knowledge, information, and different learning skills. This makes the student lose their confidence and learning motivation. When the students are a victim of demotivation, they can efficiently perform in the English learning classes, and the English education system becomes a failure. These results also agree with Adolphs et al. (2018), which highlights that when the students have to face resistance to the innovation adoption, they do not have the satisfaction that they can comprehensively understand the subject and reduce the problems in the way of learning. This reduces students' learning motivation. When the students do not take an interest in the learning processes, the English education system can be effectively run. Student demotivation mediates between resistance to innovation and the failure of the English education system.

The results indicated that students' demotivation is a mediator between the lack of intercultural communication and failure of the English education system. These results match with Gong et al. (2018), which shows that when people have no specific or appropriate communication network with people belonging to other cultures, they know a less about the way of living, their accent, behavior, and actions. Because of the lack of effective intercultural communication, they can share their ideas and attain others' knowledge. This reduces the motivation in the students about learning something belonging to another culture. When the students do not have the internal motivation to learn the English language with a particular accent because of the lack of intercultural communication, the number of students who are willing to learn the language decreases and causes failure of the English education system. These results are also in line with Kusumaningputri and Widodo (2018), which reveals that the student who lacks intercultural communication has little source of getting information regarding the second language. This shatters the learning motivation in the students, and the English learning education system cannot succeed whenever the majority of students are demotivated. The results also indicated that students' demotivation is a mediator between the lack of students' interest and failure of the English education system. These results agree with Liu (2020), students' personal interest in the subject they are going to learn affects their decisions regarding the learning resources, learning processes, tutors, hardworking, and discipline. When the students lack the interest to learn, they take ineffective decisions in learning processing and they lose their confidence and motivation, which are essential in language learning. With the presence of students without learning interest and motivation, the English education system fails.

## Implications

This study's theoretical significance is because of its contributions to the literature on education. The main focus of the study is on the causes of failure in the English education system. The study examines the influences of resistance to innovation, lack of intercultural communication, and students' lack of interest in students' demotivation. In the existing literature, one can find research on the impacts of resistance to innovation, lack of intercultural communication, and students' lack of interest in students' demotivation, but there is no simultaneous study on the nexus between these factors and students' demotivation. So, this study presents distinctive literature. Moreover, this study initiates to explore mediating impacts of student motivation on the association between resistance to innovation, lack of intercultural communication, and students' lack of interest and failure in the English education system. This study also has great empirical significance in almost all nations as it addresses a universal aspect, the education of English that is considered the mode of communication within and across the countries. This study guides the policymakers to develop the policies related to improving the English education system in China using innovation that enhances student interest and motivation. This study presents some serious causes of the student's demotivation and failure in the English education system, like resistance to innovation, lack of intercultural communication, and students' lack of interest. This study suggests that to save the English education system from failure, policies must be formulated to reduce the resistance to innovation, lack of intercultural communication, students' lack of interest, and students' demotivation. This study also guides the relevant authorities related to improve the English education system in China. In addition, this study also helps the new researchers in examining this area in future.

## Conclusion

This study was conducted to estimate the contribution of resistance to innovation, lack of intercultural communication, and students' lack of interest in students demotivation. One of its aims was to examine how the students' demotivation establishes a relation between resistance to innovation, lack of intercultural communication, and students' lack of interest and failure in the English education system. A questionnaire-based empirical survey was conducted on English language schools in China, and data regarding resistance to innovation, lack of intercultural communication, students' lack of interest, students' demotivation, and failure of the English language system. The resistance to innovation, lack of intercultural communication, and students' lack of interest have a positive association with students' demotivation. The results indicated that the resistance to adopting the innovation in education resources and processes makes it difficult for the students to gain learning skills and get disengaged from the subject. As a result, student demotivation is caused by opposition to innovation. When pupils do not have the opportunity to engage in intercultural conversation, they are unable to get insight into one another's lifestyles. This makes it difficult for students to have the urge to learn. As a result, a lack of intercultural communication demotivates students. The students who lack the learning interest are weak learning skills and demotivation. The study suggests that resistance to innovation, lack of intercultural communication, and students' lack of interest cause students' demotivation, which further takes the English education system toward failure.

## Limitations and Future Directions

The study has many limitations that are expected to be fulfilled in future theoretical research by authors. This study throws light only on the impacts of resistance to innovation, lack of intercultural communication, students' lack of interest in student demotivation, and the failure of the English education system. The institutional support, financial resources, and teachers' abilities have a crucial role in student demotivation and failure in the English education system. Future authors must also focus on these factors for protecting the English education system. In this study, students' demotivation has been addressed as a mediator between resistance to innovation, lack of intercultural communication, and students' lack of interest and failure in the English education system. In the study, demotivation affects both the independent and dependent variables; thus, it must be used as a moderator in future research. This article has taken the mediating variable and ignored the moderating effect in the model and suggested that the future studies should add moderating variable in their studies.

## Data Availability Statement

The original contributions presented in the study are included in the article/supplementary material, further inquiries can be directed to the corresponding authors.

## Author Contributions

JW contributed in introduction, literature review and methodology. LP contributed to conclusion. Both authors contributed equally to the article and approved the submitted version.

## Funding

This work was supported by Xinzhou Science and Technology Bureau Soft Science Research Project (20210501).

## Conflict of Interest

The authors declare that the research was conducted in the absence of any commercial or financial relationships that could be construed as a potential conflict of interest.

## Publisher's Note

All claims expressed in this article are solely those of the authors and do not necessarily represent those of their affiliated organizations, or those of the publisher, the editors and the reviewers. Any product that may be evaluated in this article, or claim that may be made by its manufacturer, is not guaranteed or endorsed by the publisher.
